# Bibliometric Analysis on Digital Payment Using Lens.org and Vosviewers: A Comparison of Research Between Malaysia and Poland

**DOI:** 10.12688/openreseurope.18248.1

**Published:** 2024-09-02

**Authors:** Nor Irvoni Mohd Ishar, Mior Harris Mior Harun, Azlina Hanif, Nur Arfah Mustapha, Rafal Kusa, Joanna Duda

**Affiliations:** 1 Arshad Ayub Graduate Business School, Universiti Teknologi MARA, SHAH ALAM, SELANGOR, 40450, Malaysia; 2 Faculty of Management, AGH University of Krakow, Al. Mickiewicza, Krakow, 30 30-059, Poland

**Keywords:** digital payment, Malaysia, Poland, bibliometric analysis, VOSviewer

## Abstract

*An unprecedented surge in digital payments has had a profound effect on the economic development of developing nations. This transition has enabled greater financial inclusion through the provision of banking services to populations that were previously marginalised. In addition, it has reduced reliance on physical currency, thereby improving security and transparency. This bibliometric analysis examines the research on digital payments in Malaysia and Poland, providing a comparative perspective on research patterns and contributions in these two countries. Furthermore, this study offers a comprehensive examination of the research environment in Malaysia and Poland, elucidating the distinct approaches, methodology, and areas of emphasis in each nation. These findings provide useful insights for those interested in championing digital payment initiatives, as well as contributing to a more robust and resilient digital payment framework. As digital payment ecosystems mature, their significance in altering the economic landscape of emerging countries is likely to become more evident in facilitating cross-border knowledge exchange and collaboration.*

## Introduction

The global implementation of digital payment systems is of utmost importance, especially in the context of emerging nations since it significantly impacts their economic progress. Digital payment systems cover a diverse array of technologies and platforms, which include but are not limited to mobile wallets, internet banking, contactless cards, and peer-to-peer payment applications. The period after the COVID-19 pandemic has seen an incredible transition towards digital payment methods, resulting in numerous beneficial effects on the economic conditions of developing countries.

In Malaysia, the digital payment landscape is undergoing substantial transition, owing to the growing prevalence of e-wallets, which are redefining consumer behaviour. With expected increases in e-commerce, e-wallet transactions, and digital payment user base, it is clear that digitalization is not only a continuing trend, but one that is primed for major expansion ( Khoon, 2023). Therefore, Malaysian businesses must respond to this transition in order to remain competitive. Further, the spike in e-commerce transactions, encompassing broad sectors such as fashion, groceries, and F&B services, is especially noteworthy, indicating the significant impact of e-wallets on online sales. The increasing shift to digital payments, specifically via e-wallets, represents a digital revolution in Malaysia. According to Statista (2023a), Malaysia has emerged as the second foremost nation in Southeast Asia in terms of the highest rate of adoption of cashless payment methods, where the leading e-wallet used are Touch ‘n Go, Maybank (MAE), and GrabPay.

Similarly, Poland is notable for its significant technological advancements in Europe, particularly in the use of modern non-cash payment systems. The proportion of cash transactions in the country has been consistently decreasing on a yearly basis. In 2021, a majority of 64% of the population in Poland chose to use card payments as their preferred method of transaction, while a smaller proportion of 22% continued to rely on cash-based transactions, while in the year 2022, a total of 22 million individuals from Poland actively participated in online banking, whereas 19.3 million individuals made use of mobile applications for banking purposes ( Statista, 2023b). These significant changes are due to the fast digitalization of payments, and it is projected that mobile payments would reach a value of US$ 26,893.9 million by the year 2025 ( Borcuch, 2020). Despite the observed expansion, conventional payment methods such as cash and physical credit/debit cards continue to maintain their popularity, especially among student populations ( Szumski, 2020). Nevertheless, the nation has made notable advancements in the implementation of contemporary payment modalities, emerging as the pioneer in the widespread acceptance of contactless payments and assuming a prominent role in the advancement of novel technological innovations ( Świder, 2021). This observation implies the presence of a dynamic and changing payment ecosystem in Poland, characterized by the coexistence of both conventional and new techniques. The COVID-19 epidemic brought about a significant shift in the payments sector, characterized by a sudden and considerable increase in cashless transactions during periods of lockdown. Indeed, the electronic commerce industry had significant growth, which further contributed to the widespread adoption of electronic payment methods in Poland. Among the preferred digital payment methods in Poland are Blink, Pay-by-link, and payment by card ( Statista, 2023b).

Bibliometric analysis is a widely utilized and rigorous approach for examining and evaluating substantial quantities of scientific information. It facilitates the analysis of the evolutionary intricacies within a particular discipline, while illuminating the nascent domains within this discipline. However, the utilization of this approach in the field of business research is relatively nascent and, in numerous cases, not fully developed ( Donthu *et al*., 2021). This approach has the capacity to discern prevailing research themes, temporal patterns, as well as prominent authors, institutions, and publications within domain of interest, such as digital payment. Clustering methodologies moreover have the capacity to unveil closely interconnected phrases, hence providing a nuanced comprehension of diverse facets pertaining to digital payment within the aforementioned pair of nations. Citation analysis is utilized as a method for evaluating the influence of prominent publications and identifying pivotal works that have had a substantial impact on subsequent study within the academic field. For example, Susanti and Reza (2023) conducted a study utilising bibliometric analysis to understand the change in consumer payment behaviour between 2019 to 2021. Similarly, a few other studies have also used similar approach to understand the changing trends in consumer spending patterns ( Malik & Juardi, 2023; Rozali *et al.*, 2022; Sugu & Hussain, 2021). Indeed, bibliometric approaches possess the capacity to offer a thorough depiction of scholarly literature pertaining to the domain of digital payment in Malaysia and Poland.

## Literature review

A digital payment, often known as an electronic payment, is the transfer of money from one payment account to another through the use of a digital device or channel. Digital payments can be categorized into three distinct types: somewhat digital, predominantly digital, or totally digital ( Better Than Cash Alliance, 2023), and may encompass several methods, including, e-wallet, QR codes, mobile money, as well as payment instruments like debit, credit, and prepaid cards. The growing adoption of digital banking is driven by factors such as convenience, accessibility, and time-saving benefits for customers in managing their purchases and financial services ( Kozak & Golnik, 2020b). Mobile-based digital payments enable the collection of important customer data, which facilitates analytics and market segmentation ( Khaitan & Joshi, 2023). Companies that accept digital payments can utilize this information to strengthen client acquisition and retention strategies. Furthermore, consumer data insights enable targeted marketing and personalized offerings, allowing for a more successful and specialized strategy for engaging with the audience.

The popularity of digital payments has witnessed a significant rise owing to their inherent advantages in terms of ease, security, and efficiency. Moreover, the implementation of digital payment systems can provide several benefits for firms, including enhanced financial performance, decreased instances of fraudulent activities, and the provision of instantaneous cash flow monitoring ( Charpentier, 2023). It is imperative for firms to move to digital payment systems as it facilitates the acquisition and processing of information, hence aiding in the reduction of operational expenses ( Kozak & Golnik, 2020a). Undoubtedly, an analysis of digital payment methods might yield valuable information into the factors that impede or facilitate the process of digitization. Gaining an understanding of various trends and practices will empower businesses to effectively address and reduce the digital payment divide that exists among nations. 

Comprehending the parallels and distinctions in digital payment practice between Malaysia and Poland holds significance important in various fronts. It will provide a baseline understanding for effective policy formulation and financial inclusion strategies for policymakers. A comprehensive understanding of consumer digital payment behaviors in both countries facilitates international collaboration in the advancement of technology and offers businesses valuable insights that can be used to create user-centric experiences. Moreover, this comprehension is of utmost importance in order to predict impending challenges, promote economic growth, and ensure the resilience of global digital payment ecosystems. Examining digital payment trends across various markets might also yield valuable insights on the strategies and methodologies that can propel digitalization efforts and enhance business success and ultimately, improves the capacity to navigate complexities, foster innovation, and make valuable contributions to the continuous development of digital economies on a global scale.

## Methods

The primary aim of this study is to investigate current trends in the growth of academic literature pertaining to digital payments using bibliometric analysis. It involves the evaluation of databases with a specific emphasis on indicators related with publications, such as authors, keywords, sources, geographical distributions, and others ( Dabirian *et al*., 2016). The primary data for this study was obtained from LENS.ORG, an open-source database. It is a comprehensive platform that aggregates data from various primary sources, incorporating important indicators from the original data sources to promote transparency and permit seamless interaction with other datasets. This strategy assures that the data used in the study is reliable and accessible ( Van Eck & Waltman, 2010).

An extensive search was conducted on August 27, 2023, resulting in the extraction of 107 research titles that were specifically related to digital payment. Two different search strings were used to extract data for each country. The first search string ("digital payment" OR "digital payments" AND Malaysia) yielded 95 titles and the second string ("digital payment" OR "digital payments" AND Poland) extracted 12 titles. Next, the data cleaning procedure was conducted, leading to the removal of a total of 46 titles from 107 titles. Consequently, the remained 51 datasets for Malaysia, and 10 for Poland, were subsequently analyzed using VOSviewer.

VOSviewer (vosviewer.com) is a versatile and freely available software, developed for the purpose of generating and visualizing network relationships, and assessing the interconnectedness ( Van Eck & Waltman, 2010) in bibliometric analysis. The software supports mapping citation data extracted from established databases ( Williams, 2020). By integrating the L ENS.ORG ( https://www.lens.org) database and VOSviewer, this study enhances its methodological rigor and offers a unique perspective on the developments in digital payment systems within Malaysia and Poland.

## Results and findings

The primary aim of this study is to investigate trends in the growth of academic literature pertaining to digital payments using bibliometric analysis. It involves the evaluation of databases with a specific emphasis on indicators related to publications.

### Number of publications (by document and source type)

Upon conducting a comparative analysis of the number of publications on digital payment, it becomes apparent that Malaysia significantly outperforms Poland in this regard. As depicted in Table 1, Malaysia exhibits a notable aggregate of 51 articles, signifying a robust research output. In contrast, Poland exhibits a far lower number of publications, amounting to a just 10 titles. The observed disparity implies that Malaysia exhibits a far higher quantity of research papers, potentially indicating a more expansive and varied research community, greater levels of research financing, or broader study areas of interest. Acquiring comprehension of this disparity in the aggregate quantity of publications might provide useful insights about the research landscapes and priorities of these two nations. Both Malaysia and Poland demonstrate a commendable dedication to scholarly research, as evidenced by their publication of numerous journal publications. In terms of scholarly contributions, Malaysia accounts for 71% of its total publications with 36 articles, whilst Poland represents 90% of its entire publications with 9 articles. These statistics serve to reinforce the significance of journal papers in the intellectual pursuits of both countries.

**Table 1.** Number of Publications by document and source type.

Publication Type	Malaysia	Poland
Journal Article	36 (71%)	9 (90%)
Conference Proceedings	5 (10%)	-
Book Chapter	6 (12%)	-
Others	4 (7%)	1 (10%)
Total	51	10

### Number of publications by year

Subsequently, we proceed to examine the quantity of publications on an annual basis. Upon analyzing the tabulated data depicting the aggregate quantity of publications in Malaysia and Poland over the years 2000 to 2023, several significant trends and discrepancies emerge. First and foremost, based on Table 2 it is apparent that both nations have witnessed a constant and growing trend in the volume of published works from the year 2000 to 2022. However, as of the third quarter of 2023, there has been a reduction in the number of publications for both countries. A discernible disparity is seen when examining the aggregate number of publications, with Malaysia constantly outperforming Poland.

**Table 2.** Number of Publications by Year.

Year	Total Number of Publication	
	Malaysia	Poland
2000 – 2019	5	2
2020	10	2
2021	10	2
2022	17	3
2023	9	1

In the context of Malaysia, the publication output, as measured by the quantity or number of titles, has a discernible pattern throughout the course of time. During the year 2002, a total of five titles were released, suggesting a very moderate level of productivity within that particular timeframe. Following this, between the years 2006 and 2019, a conspicuous dearth of publications was seen, indicating a period characterized by either inactivity or a deficiency in scholarly output. In the year 2020, there was a notable rise in publication activity, as seen by the release of 10 titles, signifying a renaissance in this domain. The aforementioned tendency persisted throughout the year 2021, exhibiting a consistent level of productivity with a total of ten publications. In the year 2022, there was a notable increase in the number of publications, totaling 17 titles. This surge indicates a considerable expansion in research and writing activities. Nevertheless, a marginal decline was observed in 2023, when the number of published titles amounted to nine. In general, the data exhibits fluctuations in publication production throughout time, characterized by intervals of limited or absent activity succeeded by periods of heightened productivity.

In contrast, the publication output in Poland, as measured by the quantity or number of titles, has a rather stable trend from 2019 to 2023. Prior to the year 2019, a total of two publications were released, suggesting a somewhat limited level of productivity for that particular timeframe. From 2020 to 2021, there was a continuous annual count of two titles, indicating a sustained level of publication activity. Nevertheless, a marginal rise to three titles was observed in 2022, potentially indicating an expansion or broadening of scholarly or literary pursuits. In contrast, a discernible decline was observed in 2023 (as of quarter 3), as evidenced by the publication of only one title. It is worth mentioning that the inaugural publication took place in 2017, suggesting a comparatively recent commencement of the publication chronology. In general, the data underscores a period characterized by relative stability, with a marginal upswing observed in 2022, subsequently followed by a decline in 2023. These fluctuations in publishing output across the years are prominently demonstrated.

### Most active source titles

This section highlights the journals that are favoured by scholars hailing from Malaysia and Poland. Based on Table 3, researchers from Malaysia have a significant inclination towards the "Journal of Financial Services Marketing," as seen by a frequency of 6. Furthermore, the user demonstrates a keen interest in two scholarly publications, namely the "International Journal of Academic Research in Business and Social Science" and "The Journal of Muamalat and Islamic Finance Research," with a frequency of 2 each. In contrast, researchers from Poland demonstrate a greater range of preferences. The journal "Proceedia Computer Science" is highly regarded, as seen by its frequency of occurrence, which is 2. In addition, the user demonstrates a keen interest in the "Central European Journal of Operations Research" and the "Journal of Banking and Financial Technology," with a frequency of occurrence of 1 for each publication.

**Table 3.** Three Most Active Publishing.

Source title - Malaysia	Qty	Source title - Poland	Qty
Journal of Financial Services Marketing	6	Proceedia Computer Science	2
International Journal of Academic Research in Business and Social Science	2	Central European journal of operations Research	1
The Journal of Muamalat and Islamic Finance Research	2	Journal of Banking and Financial Technology	1

When comparing the two groups of researchers, it is evident that Malaysian researchers exhibit a higher degree of focus, as a majority of them demonstrate a preference for a particular journal. On the other hand, Polish researchers exhibit a more diverse array of interests, dispersing their preferences among several academic periodicals without a prevailing preference. This observation underscores both shared characteristics, such as the inclination towards financial and academic research publications, as well as differences in the particular journals favoured by academics hailing from Malaysia and Poland.

### Citation analysis

This section reports the results for most cited articles for Malaysia and Poland. The data offers valuable insights regarding the citation frequencies of particular article titles in Malaysia and Poland, facilitating a comparative analysis of citation patterns. Table 4 and Table 5 depicts for Malaysia and Poland respectively.

**Table 4.** Most Cited Articles (Malaysia).

No	Authors (Year)	Title Source	Journal	Freq
1	Sahi, A. M., Khalid, H., Abbas, A. F., Zedan, K., Khatib, S. F., & Al Amosh, H. (2022)	The Research Trend of Security and Privacy in Digital Payment	Informatics	15
2	Norulhuda Abdullah, Fauziah Redzuan, Nor Aziah Daud (2020)	E-wallet: factors influencing user acceptance towards cashless society in Malaysia among public universities	Indonesian Journal of Electrical Engineering and Computer	12
3	Vimala Balakrishnan, Pik Yin Lok, Hajar Abdul Rahim (2020)	A semi-supervised approach in detecting sentiment and emotion based on digital payment reviews	The Journal of Supercomputing	11
4	Al Barazanchi, Israa, Haider Rasheed Abdulshaheed, Zahraa A. Jaaz, Hassan Muwafaq Gheni, Yitong Niu, Hissah Almutairi, Elika Daghighi, Shihab A. Shawkat, and Saadaldeen Rashid Ahmed. (2022)	Blockchain: The Next Direction of Digital Payment in Drug Purchase.	In 2022 International Congress on Human-Computer Interaction, Optimization and Robotic Applications	9
5	Younas, Waqar, and K. Ramanathan Kalimuthu (2021).	Telecom microfinance banking versus commercial banking: a battle in the financial services sector	Journal of Financial Services Marketing	8

**Table 5.** Most Cited Articles (Poland).

No	Authors (Year)	Title Source	Journal	Freq
1	Karol Bartkiewicz, Antonín Černoch, Grzegorz Chimczak, Karel Lemr, Adam Miranowicz & Franco Nori (2017)	Experimental quantum forgery of quantum optical money	npj Quantum Information	35
2	Anna Dewalska-Opitek, Katarzyna Bilińska, & Marek Cierpiał-Wolan (2022)	The Application of the Soft Modeling Method to Evaluate Changes in Customer Behavior towards e-Commerce in the Time of the Global COVID-19 Pandemic	Risks	1
3	Oskar Szumski (2022)	Comparative analyses of digital payment methods from the pre and post COVID-19 perspective.	Procedia computer Science	1
4	Marcin Suder, Tomasz Wójtowicz, Rafał Kusa, & Henryk Gurgul (2022)	Challenges for ATM management in times of market variability caused by the COVID-19 pandemic crisis	Central European Journal of Operations Research	1

The dataset in Table 4 illustrates the citation frequency of individual article titles by Malaysian researchers, encompassing a total of 51 articles. The paper titled "The Research Trend of Security and Privacy in Digital Payment" has garnered significant attention in the academic community, as seen by its impressive citation count of 15. This high number of citations underscores the considerable influence and relevance of the article within the topic of security and privacy in digital payment. The article titled "E-wallet: Factors Influencing User Acceptance Towards Cashless Society in Malaysia Among Public Universities" has garnered 12 citations, indicating its importance in shaping user behaviour about cashless transactions within the academic sphere. Moreover, the research paper titled "A Semi-Supervised Approach in Detecting Sentiment and Emotion Based on Digital Payment Reviews" has garnered 11 citations, indicating a significant level of attention towards sentiment analysis in the context of digital payment. The titles "Blockchain: The Next Direction of Digital Payment in Drug Purchase" and "Telecom Microfinance Banking Versus Commercial Banking: A Battle in the Financial Services Sector" have been referenced 9 and 8 times, respectively. These citations indicate the significance of these titles in the discourse surrounding the future of digital payment and the financial services sector. In general, the data highlights the significance and applicability of these papers within the field of digital payment research in Malaysia. The varying citation rates suggest varying degrees of scholarly interest.

In contrast, Table 5 presents the frequency of citations garnered by the most highly referenced works authored by Polish researchers, encompassing a total of 10 titles under consideration. The study titled "Experimental Quantum Forgery of Quantum Optical Money" has garnered considerable attention in the academic community, as evidenced by its notable 35 citations, highlighting its significant effect. In contrast, the remaining titles, namely "The Application of the Soft Modeling Method to Evaluate Changes in Customer Behavior Towards E-Commerce in the Time of the Global COVID-19 Pandemic," "Comparative Analyses of Digital Payment Methods from the Pre and Post COVID-19 Perspective," and "Challenges for ATM Management in Times of Market Variability Caused by the COVID-19 Pandemic Crisis," were cited only once. The primary article that has received the highest number of citations pertains to the topic of experimental quantum forgeries. The remaining titles of the articles seem to revolve on the examination of the effects of the COVID-19 pandemic on customer behaviour, digital payment systems, and ATM management, in that order. In general, the data suggests that the referenced publications exhibit differing degrees of influence, with one title notably surpassing the others in terms of scholarly engagement.

In summary, there are significant differences in the frequency of citations for research articles on digital payment in Malaysia and Poland. In the context of Malaysia, there exists a variation in citation rates among different titles, wherein one particular article distinguishes itself by accumulating a total of 15 citations. The research environment in Malaysia exhibits a wide range of areas of interest, encompassing security, acceptance of e-wallets, sentiment analysis, blockchain technology, and the financial services sector. In Poland, there exists a notable discrepancy in citation frequencies, particularly evident in the case of an essay on experimental quantum counterfeiting which has received a significant number of 35 citations, surpassing the attention received by other scholarly works. Each of the other papers, which examine the influence of COVID-19 on customer behavior, conduct comparative assessments of digital payment methods, and discuss the issues faced in ATM administration during the pandemic, received a citation count of only 1. The aforementioned findings highlight the disparities in impact and scholarly focus within the digital payment study fields of both nations. These disparities are indicative of the different research interests and the influence of certain issues in defining academic conversation.

### Top field of study

A field of study denotes a particular domain or specialty of scholarly or vocational concentration wherein individuals engage in endeavors such as education, research, or employment. A defined subject or branch of knowledge is encompassed within a more comprehensive academic or professional framework. With regard to academic disciplines, we have identified parallels between Malaysia and Poland.

**Figure 1.**
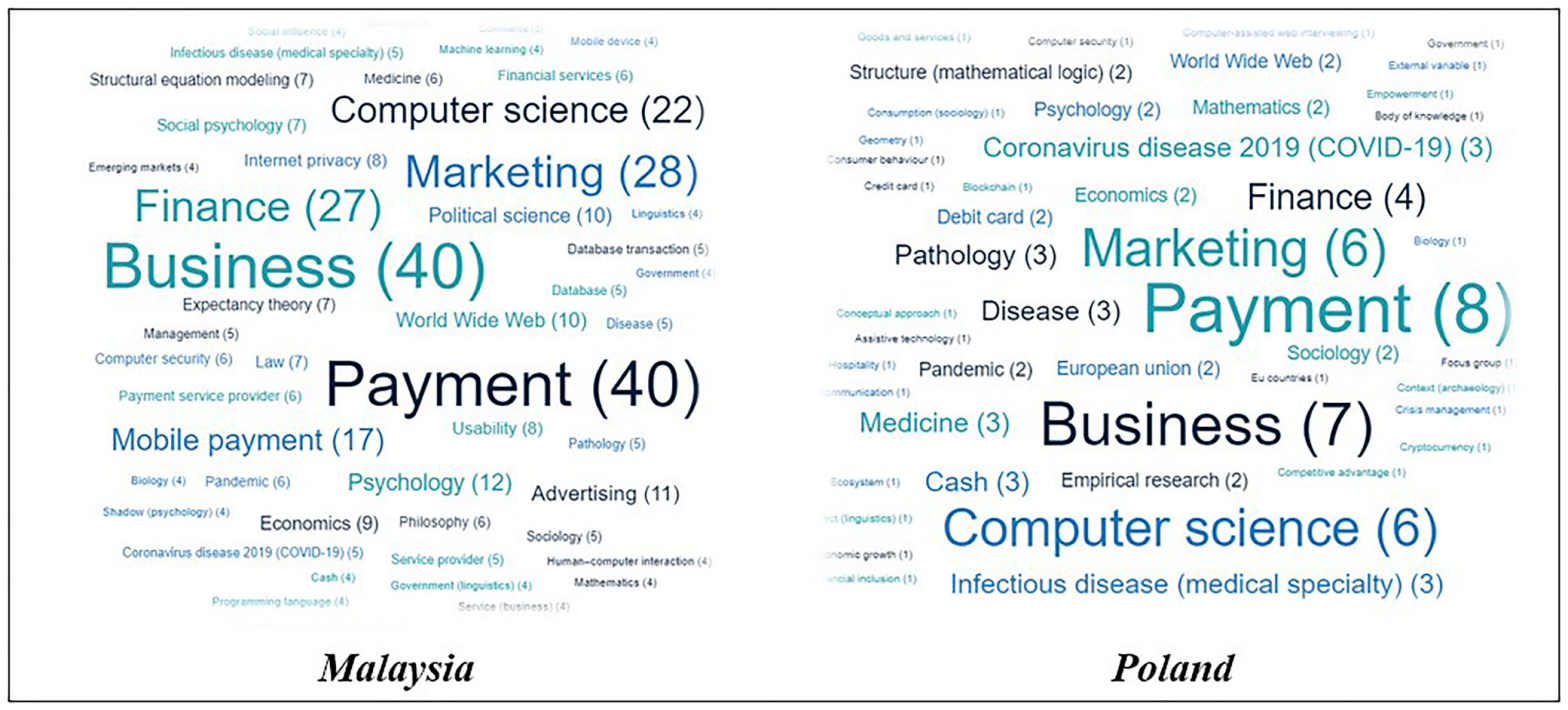
Comparison on the field of study.

Based on Figure 1, Malaysian researchers exhibit a well-distributed representation throughout several disciplines, whereby payment and business emerge as the prevailing categories, each accounting for a frequency of 40. The field of marketing exhibits a frequency of 28, whilst finance and computer-related disciplines demonstrate rates of 27 and 22, respectively. In contrast, researchers from Poland demonstrate a more focused distribution, prioritizing payment and business as their key areas of interest, albeit with lower frequencies compared to Malaysia, specifically 8 and 7, respectively. The frequencies of attention in the domains of marketing, computing, and finance are 6 and 4, respectively. This suggests that these fields have a considerably limited range of focus in comparison to researchers from Malaysia.

In conclusion, both Malaysian and Polish scholars demonstrate a shared interest in various disciplines, including payment, business, marketing, computer, and finance. In contrast, the distribution of research interests among Malaysian researchers appears to be more varied throughout different areas, whereas Polish researchers tend to have a more concentrated attention, with less variability in the frequency of their research pursuits across various fields of study. In general, the data underscores the presence of both commonalities and disparities in the domain of research interests among scholars in Malaysia and Poland.

### Research publication: co-occurrence results for Malaysia and Poland

**Figure 2.**
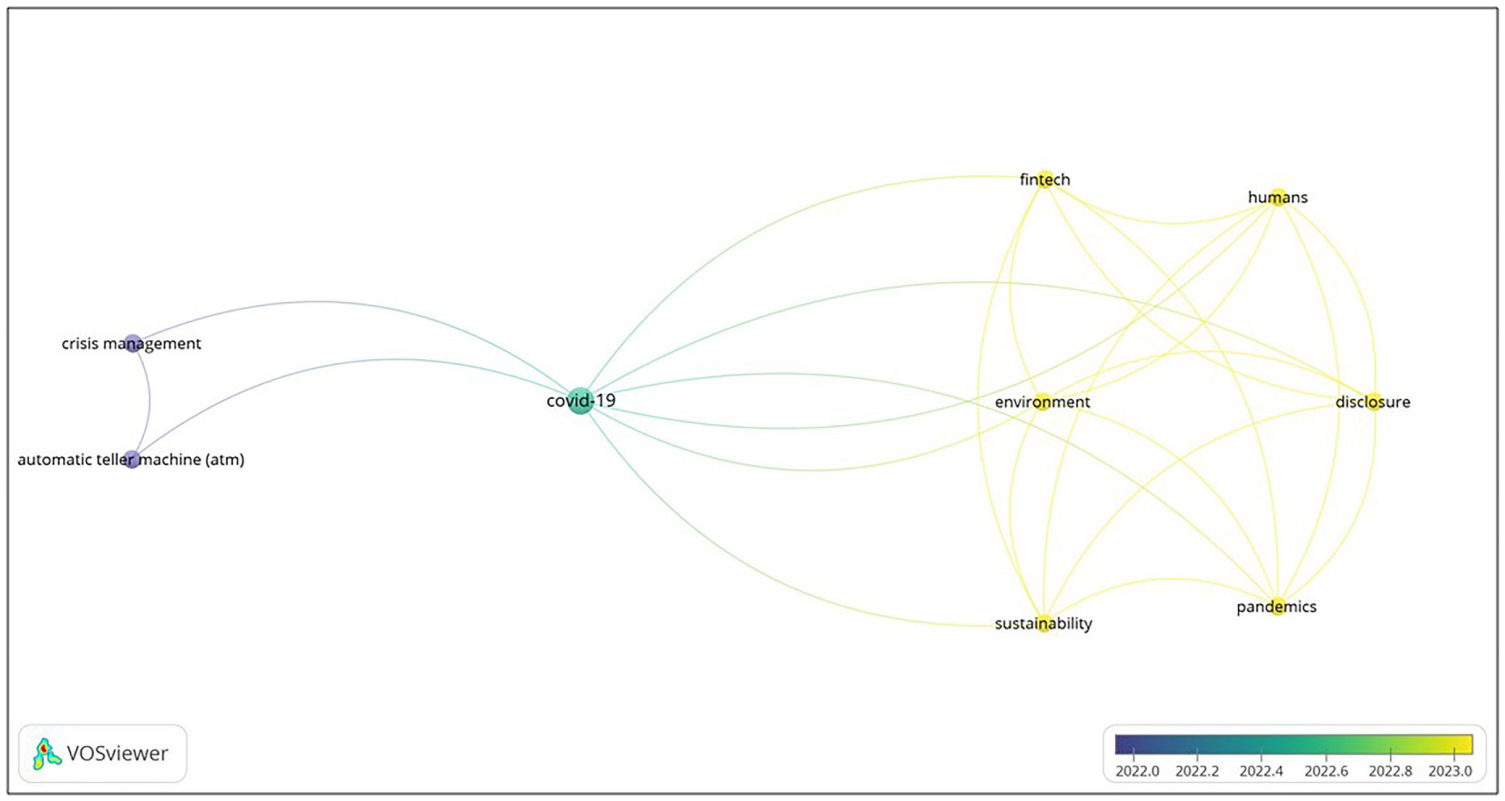
Co-occurrence map for Malaysia and Poland.

The technique of co-occurrence analysis is employed to investigate and visually represent the associations among publication in the dataset. Co-occurrence analysis is employed as a means to acquire a deeper understanding of the knowledge framework within a certain field, ascertain essential concepts, and investigate the interconnections among terms. It facilitates the comprehension of the topic framework within a corpus of literature or textual information. For this study, the researchers would like to examine the potential conceptual relationship and thematic relevance between the terminologies employed by academics from Malaysia and Poland. Figure 2 depicts the results for Malaysia and Poland. In general, two clusters have emerged from the dataset, and is connected by one central theme, Covid-19.

***Cluster 1: resilience in sustainable fintech*.** For Cluster 1, six keywords that have co-occurred for both countries are disclosure, environment, fintech, humans, pandemics, and sustainability. The identified keywords within Cluster 1 indicate a thematic concentration on the resilience of the financial technology (fintech) industry in relation to external disruptions, specifically the COVID-19 pandemic. The utilization of terminology such as "disclosure," "environment," and "sustainability" demonstrates a cognizance of the wider ramifications of fintech on societal and environmental welfare. This cluster aims to investigate the strategies employed by fintech companies in response to pandemics, their efforts to include sustainable practices, and their disclosure of pertinent information during crisis scenarios. Understanding these initiatives ensures the durability, security, and ethical considerations of digital payment systems, increasing consumer trust and adoption even during crises.

***Cluster 2: crisis management in ATM operations*.** Keywords that have co-occur are automatic teller machine (ATM) and crisis management. Cluster 2 is centered on the subject of crisis management, with a particular emphasis on the operational aspects and resilience of automated teller machines (ATMs) in times of crisis, potentially within the context of the COVID-19 pandemic. The chosen keywords suggest an analysis of the techniques and protocols that are put in place to guarantee the operational effectiveness and security of Automated Teller Machines (ATMs) in the face of difficult circumstances. This cluster explores the technological, operational, and security dimensions of crisis response in the context of ATM services. Indeed, digital payment is critical in addressing technological, operational, and security issues within the scope of crisis response, notably in the context of Automated Teller Machine (ATM) services. In the crisis response for ATM services, digital payment methods provide as a resilient solution across technological, operational, and security dimensions. These strategies could contribute to the overall success of response activities by providing alternate routes for financial services while addressing the challenges created by emergencies.

## Discussions

The results demonstrate a notable discrepancy in the quantity of publications between Malaysia and Poland, suggesting the presence of underlying causes that contribute to these disparities. The reason behind Malaysia's notable prominence in terms of publication output may be ascribed to its expansive and heterogeneous research community, which may also serve as an indicator of augmented levels of research financing. This enhanced financial support empowers researchers to engage in a greater number of studies and subsequently publish them. Moreover, Malaysia's extensive research pursuits underscore a strong academic and scientific environment spanning diverse areas of inquiry. Both Malaysia and Poland exhibit a mutual dedication to scholarly study, as seen by the fact that majority (more than 70%) of their overall publications are classified as journal articles. This statement highlights their commitment to sharing study outcomes via reputable and peer-reviewed channels, so emphasizing their adherence to rigorous and scholarly research methodologies.

An analysis of publication trends over time reveals consistent upward trajectories in the quantity of publications for both Malaysia and Poland. Malaysia's research output experiences significant growth with particularly notable increases observed from 2020 onwards. The notable increase in publication activity in 2020, as indicated by the publication of ten publications, signaling a rebound in intellectual contributions. This pattern continued in 2021, with a total of ten publications, keeping a similar level of productivity. The trend carried over into 2022, with a significant increase in the number of publications, totaling 17 titles. This increase in publication frequency indicates a renewed zeal and increased participation in the selected subject within the scientific community. In contrast, Poland’s research output had a reasonably constant development from 2019 – 2023. Prior to 2019, there were only two publications, with successive years keeping a stable annual count of two titles until 2022, when the count increased slightly to three titles. However, there was a significant drop in 2023 (as of the third quarter), with only one title published.

In the realm of academic inquiry, scholars from Malaysia and Poland demonstrate a shared inclination towards a wide range of subjects including payment systems, business studies, marketing, computer science, and finance. Nevertheless, a significant disparity becomes apparent in the allocation of research interests. Malaysian researchers have a diverse range of research interests across several domains, hence demonstrating a wider array of research endeavors. In contrast, researchers from Poland exhibit a higher level of focused concentration, with reduced fluctuation in the frequency of their research across different disciplines of study. In general, the data underscores the presence of both commonalities and unique trends in the scholarly research interests of academics in Malaysia and Poland, thereby underlining the breadth and depth of their individual fields of study.

Finally, the presence of two distinct clusters can be attributed to the multidimensional influence of exogenous shocks, particularly the COVID-19 pandemic, on several dimensions of the financial environment. Cluster 1 investigates the wider ramifications of financial technology (fintech) on sustainability and the welfare of individuals during pandemics. It emphasizes the industry's contribution to addressing environmental issues and meeting the demands of society. In contrast, Cluster 2 centers its attention on the particular obstacles and managerial approaches associated with ATM operations during periods of crisis, thereby illuminating the resilience of conventional banking infrastructure. Collectively, these clusters provide a thorough depiction of how the financial sector strategically maneuvers and adjusts to unanticipated obstacles, demonstrating a simultaneous emphasis on the overall welfare of society and the intricate operational complexities of vital financial services. The prevalent motif of resilience highlights the significance of being adaptable and well-prepared when confronted with unforeseen setbacks.

## Conclusions

In conclusion, this bibliometric analysis offers a detailed examination of the research landscape concerning digital payment in Malaysia and Poland. The findings underscore a growing fascination with digital transformation and unveil a wide range of issues and trends within this field.

The findings of this study provide useful insights for policymakers, researchers, and industry practitioners in developing strategies and initiatives to boost digital payment efforts. Stakeholders may make informed decisions and adopt evidence-based ways to support sustainable digital payment practices if they have a thorough awareness of the research landscape and emerging trends. Dive deeper into digital payment processes in various industries, such as manufacturing, services, and retail, to gain critical insights into industry-specific difficulties and possibilities in payment system digitalization.

In essence, this study adds significantly to the body of knowledge about digital payments in Malaysia and Poland. It lays the groundwork for future research, policy creation, and industry practice enhancement to support the ongoing digital revolution, notably in the field of digital payment. In the digital world, ignoring prospects for advancement in payment systems can limit competitiveness, harm operational efficiency, and represent a danger of long-term failure.

## Disclaimer

I confirm that I own all figures and tables included in my submission. The figures, created using VOSviewer, a free software, which downloaded to my computer, and the tables, based on my analysis with LENS.ORG, are my original work and findings.

## Data Availability

No data are associated with this article.

## Software availability

The software used can be freely downloaded as follows:

VosViewer via https://www.vosviewer.com

LENS.ORG via https://www.lens.org/
