# Supplementary material for: Bibliometric Analysis on Digital Payment Using Lens.org and Vosviewers: A Comparison of Research Between Malaysia and Poland
Source: Open Res Eur. 2024 Sep 2;4:191. [Version 1] doi: 10.12688/openreseurope.18248.1 (PMC11589423; doi:10.12688/openreseurope.18248.1)
Supplement: Peer review history

# Reviewer report: Reviewer response for version 1

**DOI:** 10.21956/openreseurope.19719.r45920

**Published:** 2024-12-06

**Authors:** Sabrina Leo

**Affiliations:** 1 Sapienza Università di Roma, Roma, Italy

I appreciate the opportunity to review this manuscript, which addresses the critical and timely topic of digital payments. While the paper tackles a relevant area of research, I regret to note that its theoretical and methodological contributions fall short of expectations. Below, I provide a detailed evaluation of the manuscript’s limitations and areas for improvement.

 After thoroughly reviewing the manuscript, I recognise its relevance to the ongoing global discourse on digital payment systems, particularly its comparative bibliometric analysis of Malaysia and Poland. While the study brings an interesting and timely perspective, its contributions to the academic and policy-oriented discussions appear limited by several theoretical and methodological weaknesses. That said, the manuscript does exhibit strengths that merit acknowledgment. Below, I provide a detailed evaluation of its contributions, shortcomings, and areas for improvement.

 The manuscript’s comparative approach between two distinct economies—Malaysia, as a fast-growing adopter of digital payment systems, and Poland, a European leader in contactless payment technologies—stands out as a valuable effort. By leveraging bibliometric tools such as VOSviewer and LENS.org, the study offers a methodological foundation for mapping research trends, identifying thematic clusters, and understanding citation patterns. The emphasis on contextualising findings within the broader socioeconomic realities of the two nations adds a layer of practical insight, particularly in linking digital payment adoption to economic modernisation and financial inclusion.

 The analysis of publication trends and research focus areas reveals significant disparities in the research outputs of Malaysia and Poland. This insight could inform targeted strategies for academic collaboration and policy formulation. The exploration of emerging thematic clusters, particularly around fintech resilience and ATM crisis management during the COVID-19 pandemic, provides timely reflections on the adaptability of digital payment infrastructures to external shocks.

 Despite its merits, the manuscript suffers from significant theoretical and methodological limitations that limit its contribution to the field.

 First, while bibliometric analysis is a well-established methodology, its application in this study remains superficial. The clustering of keywords, for example, is underdeveloped and fails to provide deeper insights into the underlying themes and interconnections. For instance, the paper identifies clusters related to fintech resilience and ATM crisis management but does not elaborate on the broader implications for the digital payment ecosystem or policy recommendations. The analysis remains descriptive rather than offering actionable insights.

 Second, the manuscript does not sufficiently address the limitations of its bibliometric data. The disparity in research outputs between Malaysia and Poland is highlighted, but the underlying reasons—such as differences in academic funding, research priorities, or institutional capacities—are not explored. Additionally, the heavy reliance on data extracted from a single database (LENS.org) raises concerns about the comprehensiveness and representativeness of the dataset. A more rigorous cross-validation using other bibliometric databases such as Scopus or Web of Science would have strengthened the study’s reliability.

 Moreover, the selection criteria for publications are inadequately justified. While the exclusion of certain studies is mentioned, there is no discussion of the potential bias introduced by these exclusions or their impact on the findings. This omission detracts from the transparency and robustness of the methodology.

 Another significant omission in the manuscript is the lack of a dedicated section reviewing existing literature reviews on digital payments. A robust bibliometric study should contextualise its findings within the broader academic landscape, and this requires engaging with prior literature reviews that have shaped the field. Notable examples include studies such as Dahlberg et al. (2008) and Dahlberg et al. (2015), which provide foundational insights into mobile payments and their adoption. Similarly, more recent works such as Alkhowaiter (2020), Shaikh and Karjaluoto (2015), and Panetta et al. (2021) explore critical themes such as technological adoption, user behaviour, and the evolution of digital payment systems.

 Incorporating an analysis of these reviews would not only position the current manuscript within the context of existing scholarship but also help in identifying gaps that this study aims to address. By neglecting this aspect, the manuscript misses an opportunity to demonstrate its added value and differentiate its contributions from those of previous studies. I strongly recommend including a section that systematically analyses these influential reviews, thereby enhancing the paper's theoretical grounding and academic rigour.

 From a theoretical perspective, the manuscript lacks a clear conceptual framework to contextualise its findings. While it provides a comparative analysis of research output, it does not integrate this with existing theoretical discussions on digital payments, such as those related to financial inclusion, consumer behaviour, or technological adoption frameworks. Without such a foundation, the study’s contributions to academic discourse remain limited.

 Finally, the study’s policy implications are underdeveloped. While it identifies themes and trends in research, it does not effectively translate these findings into actionable recommendations for policymakers, particularly in the areas of digital payment adoption, financial inclusion, or cross-border collaboration.

 While the manuscript offers a novel comparative lens on the research landscapes of Malaysia and Poland, it ultimately falls short in advancing the academic and practical understanding of digital payment systems. The study’s descriptive focus, methodological gaps, and lack of theoretical integration diminish its impact.

 To enhance their contribution, the authors should consider expanding the bibliometric dataset to include multiple sources and ensure greater representativeness. This would allow for a more detailed and critical analysis of the identified clusters, linking them to broader theoretical frameworks and practical implications. It would also strengthen the discussion of contextual factors that influence research output in Malaysia and Poland, such as funding disparities or policy differences, and develop clear and actionable recommendations for policymakers and stakeholders based on the findings.

 In its current form, the manuscript highlights important trends but does not sufficiently elevate them to the level of meaningful academic or policy contributions. I recommend significant revisions to address the aforementioned issues before considering publication.

If applicable, is the statistical analysis and its interpretation appropriate?

Partly

Is the study design appropriate and is the work technically sound?

Partly

Is the work clearly and accurately presented and does it engage with the current literature?

Partly

Are the conclusions drawn adequately supported by the results?

Partly

Are sufficient details of methods and analysis provided to allow replication by others?

Partly

Are all the source data and materials underlying the results available?

Partly

Reviewer Expertise:

Sabrina Leo is Associate Professor at the Department of Management, Faculty of Economics, Sapienza University of Rome. She holds a Ph.D. in Banking and Finance from the same institution. With extensive teaching experience across various financial disciplines, she currently lectures on courses such as Economia degli Intermediari Finanziari and Strategie, Performance e Digital Innovation nelle Banche. Her research interests include digital innovation in banking, digital payments, sustainable finance, and ESG metrics, with a notable focus on Distributed Ledger Technology and blockchain applications in the financial sector.Professor Leo has published extensively on these themes, including contributions that explore blockchain integration in ESG reporting (https://doi.org/10.1080/09537325.2021.1999921), credit guarantee schemes (https://doi.org/10.1007/978-3-031-67431-0_30), and the broader implications of DLT for banking efficiency and resilience(https://doi.org/10.1017/9781009411783). She is a prolific author, contributing to leading academic journals and serving as editor for books on contemporary financial issues. Professor Leo has secured numerous competitive research grants, including those addressing ESGdisclosures and blockchain innovation. Recognized for her academic excellence, she received the "Outstanding Woman Researcher in Banking and Finance" award at the VIWA 2022.

I confirm that I have read this submission and believe that I have an appropriate level of expertise to state that I do not consider it to be of an acceptable scientific standard, for reasons outlined above.

## References

[1] : Digital payment and banking adoption research in Gulf countries: A systematic literature review. *International Journal of Information Management* .2020;53: 10.1016/j.ijinfomgt.2020.102102 10.1016/j.ijinfomgt.2020.102102

[2] : Past, present and future of mobile payments research: A literature review. *Electronic Commerce Research and Applications* .2008;7(2) : 10.1016/j.elerap.2007.02.001 165-181 10.1016/j.elerap.2007.02.001

[3] : A critical review of mobile payment research. *Electronic Commerce Research and Applications* .2015;14(5) : 10.1016/j.elerap.2015.07.006 265-284 10.1016/j.elerap.2015.07.006

[4] : Mobile banking adoption: A literature review. *Telematics and Informatics* .2015;32(1) : 10.1016/j.tele.2014.05.003 129-142 10.1016/j.tele.2014.05.003

[5] : The development of digital payments – Past, present, and future – From the literature. *Research in International Business and Finance* .2023;64: 10.1016/j.ribaf.2022.101855 10.1016/j.ribaf.2022.101855

# Reviewer report: Reviewer response for version 1

**DOI:** 10.21956/openreseurope.19719.r45915

**Published:** 2024-11-25

**Authors:** Marija Antonijević

**Affiliations:** 1 Institute of Economic Sciences, Belgrade, Serbia

The introduction section needs better structuring and should include the following elements:

 1. What is the significance of the research topic?

 2. What were the findings of prior studies?

 3. What areas require further research (research gaps)?

 4. What is the research objective? The aim and what the authors want to examine should be clearer and more concise.

 5. What makes this research innovative and unique (novelty)? The authors should explain why Poland and Malaysia were chosen for examination.

 It is necessary to strengthen the literature review section and include the results of previous similar studies related to digital payments.

 It is unnecessary to state the research aim in the Methods and Results and Findings sections. The results and findings section should be entitled “Results”. The authors should adequately position tables and figures to align with the text.

 Tables 3, 4, and 5 should not be placed consecutively. Thus, there should be explanatory text between them. Additionally, the authors should avoid repeating data from the tables and focus only on interpretation.

 The discussion section is missing a comparison of the results with other studies. The paper lacks limitations and directions for further research. It is unclear how this study specifically contributes to policymakers.

If applicable, is the statistical analysis and its interpretation appropriate?

Partly

Is the study design appropriate and is the work technically sound?

Yes

Is the work clearly and accurately presented and does it engage with the current literature?

Partly

Are the conclusions drawn adequately supported by the results?

Partly

Are sufficient details of methods and analysis provided to allow replication by others?

Partly

Are all the source data and materials underlying the results available?

Partly

Reviewer Expertise:

Digital banking, Financial inclusion, Digital financial inclusion

I confirm that I have read this submission and believe that I have an appropriate level of expertise to confirm that it is of an acceptable scientific standard, however I have significant reservations, as outlined above.

# Reviewer report: Reviewer response for version 1

**DOI:** 10.21956/openreseurope.19719.r45062

**Published:** 2024-11-21

**Authors:** Santanu Purohit, Dr Sujoita Purohit

**Affiliations:** 1 University of Petroleum & Energy Studies, Dehradun, Uttarakhand, India; 2 Department of science, Mohanlal Sukhadia University (Ringgold ID: 29798), Udaipur, Rajasthan, India

**Importance of this manuscript for the scientific community:**

 Given the dynamic nature of technology enhancement and its specific technology-related operational requirements in digital payment, this manuscript substantially contributes to the scientific community by discussing the usage of and challenges related to the subject.  I find that the comprehensive way of targeting this concept makes for an overall valuable contribution to the manuscript.

**Suitability of the title of the article :**

 The title, which succinctly encapsulates the digital payment aspect in both countries, is not only fitting but also enhances the manuscript's readability and alignment with its subject matter.  

**The comprehensiveness of the abstract:**

 The article comprehensively captured the topic from both Malaysia's and Poland's perspectives. While the analysis is done beautifully, various sectors responding to digital payment should have been brought together to give a holistic impact of the digital payment transformation in society in both countries, and a comparison could have been handy.

**Appropriateness of the subsections and structure of the manuscript:**

 The manuscript's structure and subsections make sense, and the subsections flow together logically. The thought evolution is straightforward for readers to follow because each segment is properly identified.  

**Scientific correctness of this manuscript:**

 The manuscript's thorough analysis of Digital payment from both countries' perspectives further supports its technical soundness. The text is scientifically reliable and beneficial for both scholars and industry personnel because of its systematic analysis and application of evidence-based findings.

**Sufficient references covered in the manuscript:**

 The references include relevant sources, which are often sufficient. However, adding more current references (within the last two to three years, for example) that highlight digital payment in various sectors in the countries and technical developments in customer service could improve the literature's foundation.  

**Suitability of the language/English quality of the article for scholarly communications:**

 The quality of the English and the language are appropriate for academic communication. Technical principles are successfully communicated in the well-written manuscript's straightforward and succinct language. While minor sentence structure adjustments could increase readability even further, the manuscript's language quality is generally suitable for professional and academic audiences.

If applicable, is the statistical analysis and its interpretation appropriate?

Partly

Is the study design appropriate and is the work technically sound?

Yes

Is the work clearly and accurately presented and does it engage with the current literature?

Yes

Are the conclusions drawn adequately supported by the results?

Yes

Are sufficient details of methods and analysis provided to allow replication by others?

Yes

Are all the source data and materials underlying the results available?

Yes

Reviewer Expertise:

digital transformation, service marketing, marketing management

We confirm that we have read this submission and believe that we have an appropriate level of expertise to confirm that it is of an acceptable scientific standard.

# Reviewer report: Reviewer response for version 1

**DOI:** 10.21956/openreseurope.19719.r45069

**Published:** 2024-11-08

**Authors:** Weilun Huang

**Affiliations:** 1 Wenzhou Business College, Wenzhou, China

The study titled *"Bibliometric Analysis on Digital Payment Using Lens.org and VOSviewer: A Comparison of Research Between Malaysia and Poland"* offers a comparative bibliometric analysis of digital payment research between Malaysia and Poland. Utilizing the Lens.org database and VOSviewer for visualization, the authors examine trends, influential publications, and the structure of research outputs in digital payment studies within these two countries. The study identifies key themes like resilience in fintech and crisis management in ATM operations, highlighting each nation's unique focus and challenges in digital payment.

 Detailed Review Based on Criteria

1. **Is the work clearly and accurately presented and does it engage with the current literature?**
**Response:** Partly
**Evaluation:** The article presents a substantial amount of data on the bibliometric analysis of digital payment research in Malaysia and Poland. However, it occasionally lacks clarity in distinguishing between purely descriptive data and analytical insights that connect this research to broader trends in digital payment. While the authors reference some recent literature, the review could be enhanced by integrating additional global perspectives on digital payment evolution to situate the findings within a broader context.
**Recommendation:** The authors should deepen their engagement with recent and global literature on digital payments, especially works on fintech resilience and digital transformation post-COVID-19, to underscore the relevance of the study within a global framework.
2. **Is the study design appropriate and is the work technically sound?**
**Response:** Yes
**Evaluation:** The study utilizes a recognized approach—bibliometric analysis with tools like Lens.org and VOSviewer, ensuring technical robustness. The use of quantitative indicators and network visualizations through VOSviewer provides a credible methodological foundation, appropriate for uncovering publication patterns and co-occurrence of research themes.
**Recommendation:** To further strengthen the study design, the authors might briefly discuss the limitations of bibliometric analysis, such as biases in database coverage or the exclusion of certain types of publications that may impact the findings.
3. **Are sufficient details of methods and analysis provided to allow replication by others?**
**Response:** Partly
**Evaluation:** While the methods section outlines the data sources, search terms, and software tools used, it lacks specifics on certain analytical decisions, such as threshold settings in VOSviewer or criteria for dataset cleaning. These omissions may make replication challenging for other researchers.
**Recommendation:** The authors should include more granular details on the methodological steps, particularly any filtering criteria and parameter settings in VOSviewer. Such additions will ensure transparency and facilitate replication.
4. **Are all the source data and materials underlying the results available?**
**Response:** Partly
**Evaluation:** While the main source, Lens.org, is open-access, the article does not specify if the specific datasets extracted are openly accessible or if they are available upon request. Given that data transparency is essential for replicability, this aspect should be clarified.
**Recommendation:** The authors should consider including an appendix or a link to the datasets or a clear statement on data availability to enhance transparency.
5. **If applicable, is the statistical analysis and its interpretation appropriate?**
**Response:** Partly
**Evaluation:** The article uses citation analysis as a measure of influence, but the interpretation could benefit from a more nuanced statistical perspective, particularly regarding the significance of citation disparities between the two countries. This would provide a more detailed analysis of why certain trends are statistically meaningful.
**Recommendation:** Adding statistical tests or significance measures when comparing citation rates or publication trends could provide deeper insights into the observed patterns.
6. **Are the conclusions drawn adequately supported by the results?**
**Response:** Partly
**Evaluation:** The conclusions generally align with the results presented; however, they could benefit from a more critical discussion. For example, the article notes Malaysia’s higher output but does not thoroughly discuss potential factors or systemic influences (e.g., economic or policy differences) that might account for this.
**Recommendation:** The authors should offer a more comprehensive analysis of the implications of their findings, especially in terms of how digital payment research can impact policymaking or industry practices in each country.

Summary of Recommendations to Improve Scientific Soundness

1. **Literature Engagement:** Broaden the literature review to incorporate recent global studies on digital payments and fintech resilience.
2. **Methodological Detail:** Provide specific settings and criteria for data extraction and visualization to enable replication.
3. **Data Transparency:** Ensure dataset accessibility or clarify data availability in the article.
4. **Statistical Rigor:** Introduce statistical tests to strengthen interpretation of citation patterns and publication trends.
5. **Analytical Depth in Conclusions:** Expand the discussion on the implications of national research disparities, possibly including socio-economic factors.

The other suggestions are:

 1. Logical Structure

**Assessment**: The logical structure across sections lacks cohesion, particularly in linking the theoretical foundation to the empirical results. While the article provides abundant comparative data on digital payment research in Malaysia and Poland, there is an absence of organic transitions between sections, making it challenging for readers to understand the interrelationships among each research focus.

**Suggestion**: To improve logical coherence, consider reorganizing the sections. In the theoretical foundation, include an overview of digital payments in the global context to better introduce the cases of Malaysia and Poland. Then, connect the theoretical framework to the empirical results through clear paragraph transitions, forming a causal relationship between the two. Additionally, include brief summaries at the end of each section to emphasize key points and set up the following section.

 2. Theoretical Foundation

**Assessment**: The theoretical foundation references some classic literature on digital payments and fintech; however, it does not fully discuss the economic and social impacts of digital payments, nor does it reflect how the theoretical basis supports the empirical analysis. Moreover, the current section on theory mainly focuses on the global development of digital payments and lacks an in-depth analysis of the specific contexts of Malaysia and Poland.

**Suggestion**: The theoretical foundation should focus more on core issues such as how digital payments impact economic development and financial inclusion, citing more diverse and recent literature. Include specific socioeconomic contexts of Malaysia and Poland, as well as the influence of related policies on digital payment development, to provide a solid theoretical basis for the empirical analysis. Additionally, consider supplementing the discussion on the impact of fintech on cross-border knowledge sharing to support the comparative parts of the paper.

 3. Empirical Results

**Assessment**: The empirical results section presents substantial bibliometric data but lacks depth in analysis, resulting in relatively superficial conclusions. This section mainly reports data without discussing the underlying reasons or possible explanations for the findings. For example, the significant difference in publication volume between Malaysia and Poland is noted, but the paper does not explore potential institutional, cultural, or economic factors.

**Suggestion**: Increase the depth of interpretation within the empirical results section. For instance, analyze differences in digital payment policies, the degree of fintech adoption, and consumer acceptance levels between Malaysia and Poland to explain the statistical differences observed in the bibliometric analysis. Using charts or models to demonstrate how different factors influence digital payment research in each country could further enhance this section. Additionally, consider incorporating statistical tests, such as significance testing on various variables, to further validate differences in trends, citation rates, and other aspects between the two countries.

 4. Recommendations

**Assessment**: The recommendations for improving digital payment research are relatively general and do not provide specific measures linked to the empirical findings. Furthermore, some recommendations lack a strong connection to the preceding empirical data, making them less relevant to the specific issues uncovered in the study.

**Suggestion**: The recommendations section should be closely linked to the findings of the empirical results. For instance, if the results show a higher volume of literature in Malaysia compared to Poland, the suggestions could address policy guidance, research funding, and other measures that Poland could adopt to strengthen its digital payment research. It is also advisable to expand on the limitations of the research, such as the constraints of different data sources and the applicability of bibliometric methods, to help readers understand the reliability and generalizability of the current findings. Further, consider proposing directions for future research, such as the potential benefits of cross-border collaboration and region-specific policy experimentation, to provide more targeted guidance for researchers and policymakers.

If applicable, is the statistical analysis and its interpretation appropriate?

Partly

Is the study design appropriate and is the work technically sound?

Yes

Is the work clearly and accurately presented and does it engage with the current literature?

Partly

Are the conclusions drawn adequately supported by the results?

Partly

Are sufficient details of methods and analysis provided to allow replication by others?

Partly

Are all the source data and materials underlying the results available?

Partly

Reviewer Expertise:

Digital Payment

I confirm that I have read this submission and believe that I have an appropriate level of expertise to confirm that it is of an acceptable scientific standard, however I have significant reservations, as outlined above.
